# The Physiology of Opinion

**Published:** 1906-01-13

**Authors:** 


### The Physiology of Opinion.

We constantly hear it asserted in modern life, by very well-meaning people, that they "think" so-and-so about some current question, which may often be of a somewhat abstruse character, requiring the collection and investigation of many and diverse data as the only possible foundation of a sound judgment with regard to it. Still more have arrived at "opinions" which they cherish, and any opposition to which provokes a feeling of automatic or instinctive hostility; a feeling which may go far to explain the recent endeavours of certain "Liberal" politicians to refuse a fair hearing to their opponents. An opinion, be it noted, is "a persuasion of the mind in the absence of certain knowledge," and hence the American platform speaker, who finished his discourse by declaring, "These are my opinions, but if you don't like 'em they can be changed," was really far more worthy of respect than many of those by whom his readiness to bend to circumstances has been condemned. In truth, the formation of what are called opinions is a very curious process, and one which, because it has been regarded as falling within the province of the metaphysician, has never hitherto received due attention from the physiologist. The verbiage of the former may now, perhaps, with general consent be consigned to the rubbish heap of the past, and the nature of thought may be investigated on the same lines, and by the same methods, as the nature of sensation. If this be so, it is not extravagant to suppose that the reception or in some cases the original formation of an idea is a function of a certain type of nerve-cell, and that the phenomena of association, and consequently of reflection, are the results of the transmission of impressions from the cell originally influenced to others which are in relation with it through its fibres. Malebranche, more than two centuries ago, suggested that certain "paths of conduction" were formed by the frequent recurrence of the same associations, and that nervous impulses traversed these paths with increased facility; and his hypothesis has, on the whole, derived material support from modern researches. Sometimes people are born in whom certain paths of conduction seem to be congenital, insomuch that they are able to perform, without difficulty and almost without instruction, intellectual acts which ordinary persons only learn to accomplish with difficulty and as a result of continuous effort. Such favoured people are correctly described as examples of "genius," and are exemplified by born musicians, born calculators, born mathematicians, and the like. The average man has to form all his paths of conduction for himself, more or less aided by instruction and opportunity; and, in adult age, his powers of mental activity are practically limited by the extent, the variety, and the character of his "path formations" in earlier life. This extent and character, in the great majority of people, are very limited; and the immense mass of mankind, although capable of exercising sound judgment in matters within the spheres of their habitual activities, are totally at a loss beyond them, and can only fall back upon phrases which they have learnt by rote. The intelligence of the average man may be compared to a neglected garden, through which, at one period, it would have been easy to walk and to make paths in any direction, but which, after a time, becomes choked up and impassable except in the directions in which paths have been actually made, and have been maintained by constant traversing. It is thus that people who may be really very correct and acute in things familiar to them are liable to become "fossilised," and to rest contentedly upon past beliefs or upon long exploded errors, without even the power of paying due attention to the changes which time has wrought. There is no path in their brains that way, and they are too old to make a new one. The very effort, or even the very invitation to do so is painful to them, and often excites them to anger rather than to reflection. The mischief is that they do not, as a rule, in the least degree recognise their own limitations and incapacities; and hence they either strenuously argue about matters as to which they have never possessed the power of reasoning, or they furiously reiterate verbal formulas which have lost their applicability to the existing conditions of the controversy. They would do better, as a rule, to accept and act upon the teaching of some master of the subject matter at issue; and to admit their own incapacity to be anything more than his disciples.

